# Safety and Performance Characteristics of Outpatient Medical Thoracoscopy and Indwelling Pleural Catheter Insertion for Evaluation and Diagnosis of Pleural Disease at a Tertiary Center in Canada

**DOI:** 10.1155/2017/9345324

**Published:** 2017-08-30

**Authors:** Robert Kyskan, Pen Li, Sunita Mulpuru, Carolina Souza, Kayvan Amjadi

**Affiliations:** ^1^Department of Medicine, Division of Respirology, University of British Columbia, Vancouver, BC, Canada; ^2^Department of Medicine, Division of Respirology, University of Alberta, Edmonton, AB, Canada; ^3^Department of Medicine, Division of Respirology, University of Ottawa, Ottawa, ON, Canada; ^4^Department of Radiology, University of Ottawa, Ottawa, ON, Canada

## Abstract

**Background:**

Many centers performing medical thoracoscopy (MT) to diagnose pleural disease will insert a chest tube and admit patients to hospital after the procedure, which is inconvenient for patients and contributes to healthcare costs. We report the data on the safety, outcomes, and performance characteristics of outpatient MT with indwelling pleural catheter (IPC) insertion in a large Canadian cohort.

**Methods:**

This retrospective cohort study reviewed patients who underwent outpatient MT and IPC insertion under conscious sedation. Patients without complications were discharged the same day. We report the data on safety, outcomes, and performance characteristics of our program.

**Results:**

Outpatient MT and IPC insertion was performed on 218 patients. 99.1% of patients were safely discharged the same day. There was no procedure associated mortality. Pleural malignancy (59.6%) and nonspecific pleuritis (29.4%) were the most common pathologies. Pleural nodularity detected endoscopically was excellent at predicting malignancy with a positive predictive value of 92.5% and is more frequently detected endoscopically when compared to CT scan (*p* < 0.001).

**Conclusions:**

In the setting of a comprehensive pleural disease program, outpatient MT can be safely performed and is an alternative to an inpatient surgical approach for undiagnosed pleural effusions.

## 1. Introduction

Medical thoracoscopy (MT) is a minimally invasive procedure that utilizes a semirigid pleuroscope in order to visualize the pleural space and perform biopsies for diagnostic purposes in patients with pleural disease. Pleural fluid analysis can only establish the diagnosis in approximately 75% of cases overall and 60% of malignant effusions [1]. Historically, closed pleural biopsies have been performed in this setting, but this has proven less sensitive than MT [2].Video-assisted thoracoscopic surgery (VATS) can be performed for undiagnosed effusions or pleural disease, but this option is more invasive and requires operating room time. Furthermore, many centers performing either MT or VATS insert a chest tube and admit patients to hospital after the procedure, which is less convenient for the patient and could be associated with higher healthcare costs. Studies have demonstrated that MT is safe [3]. One study also suggested that MT performed as an outpatient is safe and feasible when performed in the appropriate patient population [4]. As such, MT is recommended by international guidelines [1] and is increasingly performed. Our outpatient MT program was established on December 2007, at our tertiary care hospital, as an alternative to surgery for investigating undiagnosed pleural effusions. We combined MT and indwelling pleural catheter (IPC) insertion, as IPCs are effective in treating malignant effusions, even in the setting of trapped lung and facilitates outpatient management [5]. We report the data on the safety, outcomes, and performance characteristics on our MT program.

## 2. Methods

### 2.1. Study Design and Setting

We performed a retrospective cohort study including all adult patients who underwent planned outpatient MT for assessment of pleural disease between December 2007 and February 2014 at The Ottawa Hospital (TOH), with a follow-up period of at least 2 years. Our study was approved by the Ottawa Health Sciences Research Ethics Board. TOH is a tertiary care academic hospital with 1100 beds and services a catchment area of approximately 1 million people.

### 2.2. Study Population

We included all consecutive patients who were referred to our outpatient pleural effusion clinic who underwent outpatient MT. Patients were referred from the local city or adjacent rural areas. All patients were evaluated by an interventional pulmonologist and had undiagnosed pleural disease or have confirmed metastatic cancer and required additional tissue for cancer characterization and mutational analyses. Contraindications to MT included absence of a pleural space, irreversible bleeding diathesis, hemodynamic instability, or evidence of pleural infection. Procedural informed consent for MT was obtained in all patients.

### 2.3. Data Collection and Outcomes

Study variables were collected from patient charts, using a standardized chart abstraction instrument. We collected several patient and procedural related variables including age, gender, Eastern Cooperative Oncology Group (ECOG) performance status, baseline dyspnea index (BDI), transition dyspnea index (TDI) two weeks after procedure, number of prior thoracenteses, computed tomography (contrast or noncontrast) imaging results, procedural duration, medication used for conscious sedation, volume of pleural fluid drained, pleural fluid analysis, endoscopic findings, histologic diagnosis, need for additional diagnostic procedures, complications, and time to IPC removal. The primary outcome was the safety of performing outpatient MT as measured by rates of complications and need for admission after procedure. Secondary outcomes included need for repeat diagnostic procedure, need for repeat pleural diagnostic procedure, and patient symptom scores as measured by TDI after procedure. The BDI and TDI were measured in patients to track symptomatic improvement. A BDI of less than 6 reflects severe dyspnea, and the minimal clinically significant TDI is one [6].

### 2.4. Clinical Practice

All procedures were performed in the endoscopy suite. After positioning the patient in the lateral decubitus position with the affected side up, an appropriate entry site was marked using bedside ultrasound guidance. The patient was connected to cardiac, blood pressure, and pulse oximetry monitors. Moderate conscious sedation with midazolam and fentanyl was administered. The patient continued breathing spontaneously and was provided with supplemental oxygen provided by nasal cannula as needed. The skin was cleaned and the patient draped in sterile fashion. 10–15 mL of 1% lidocaine solution was used to anesthetize the planned entry site, and a small incision was made. Kelly forceps were then used to blunt dissect to the pleural space and an 8 mm disposable trocar was inserted. A semirigid pleuroscope (Olympus LTF-160) was then inserted through the trocar and all the pleural fluid was aspirated. The pleural cavity was inspected with the exception of the lung apex. Any parietal pleural abnormalities were biopsied. Random biopsies of the posterior parietal pleura were performed in the absence of visible abnormalities. Biopsies were sent for pathology and for microbiology including acid fast bacilli. At the end of the procedure, an IPC (PleurX) was subsequently inserted and connected to a water seal suction device at −20 cm H2O pressure, followed by −40 cm H2O pressure. The patients were disconnected from suction once no further air leak was noted in the underwater seal despite intentional cough. Postprocedural chest X-rays were performed immediately off suction and two hours after to confirm lung reexpansion. Discharge criteria included observation for at least two hours, adequate pain and nausea control, oxygen saturations returned to baseline or improved, hemodynamic stability, and absence of pneumothorax or stable pneumothorax on two-hour postprocedure chest X-ray. After two hours of observation, patients without significant complications were discharged with oral analgesic.

Home care nursing was arranged to perform drainages three times per week, and all patients followed up in the pleural effusion clinic in two weeks. Subsequent follow-up was arranged every six to eight weeks. The IPC was kept in place until drainages were less than 50 mL with two consecutive drainages, and there was no increase in pleural effusion size on chest X-ray. At IPC removal, the skin was cleaned and draped in a sterile fashion. 10 mL of lidocaine 1% was used to anesthetize the insertion site, and the IPC was dissected out and removed.

Repeat procedures were performed if the suspicion of malignancy was still high despite negative pleural biopsies. The chosen procedure was based on clinician assessment and may include CT-guided needle biopsies, surgical VATs, or bronchoscopy with endobronchial ultrasound.

### 2.5. Statistical Analysis

We used means, medians, standard deviations, and interquartile ranges to describe continuous variables and proportions to describe categorical variables. Comparisons between categorical variables were made with *X*^2^ with *p* < 0.05 indicating significance. Correlations were calculated using the Pearson correlation coefficient, with values > 0.6 representing “strong correlation” and values > 0.8 representing “very strong correlation.”

## 3. Results

### 3.1. Patient Characteristics

A total of 218 outpatient MT and IPC insertions were completed between December 2007 and February 2014 at TOH. The mean age (±SD) was 68.2 (±12.5) years. Refer to Table 1 for details. The largest proportion of patients presented with ECOG performance status of 2 at 48.6%. However, 33.5% of the patients had ECOG ≥ 3. A total of 85.3% of patients had undergone at least 1 thoracentesis previously, which failed either to yield a diagnosis or to provide adequate cell block for all testing required. The most common imaging abnormality identified on CT scan was pleural thickening seen in 41.1% of patients followed by pleural nodularity in 31.8%. There were 11.9% of patients who did not have a CT chest within 3 months prior to their procedure (Table 1). The mean BDI was 3.7 ± 1.3 and mean TDI was 5.6 ± 2.0, indicating symptomatic improvement.

Procedural details are summarized in Table 2. The mean procedural duration was 46.7 ± 13.6 minutes. Patients received conscious sedation with mean doses of midazolam 2.2 ± 0.6 mg and fentanyl 91.6 ± 40.4 mg. The mean total amount of pleural fluid drained was 1513 ± 1054 mL. A strong positive correlation between TDI and volume of pleural fluid drained was identified with a Pearson coefficient of 0.69. The most common endoscopic findings included pleural thickening (58.6%), pleural nodularity (48.6%), and adhesions (43.6%). There were 10 patients (4.6%) with no endoscopic abnormalities identified.

Pathology findings are outlined in Table 3. Pathologic confirmation of malignancy was found in 130 patients (59.6%). Non-small-cell lung cancer was the most common malignancy with 47 patients (21.6%) and 42 of those patients were classified as adenocarcinoma. Mesothelioma was diagnosed in 20 (9.2%) of cases. Findings were classified as nonspecific pleuritis (NSP) in 64 (29.4%) of cases with 9 (4.1%) of the procedures demonstrating atypical mesothelial or cellular changes that could not be further classified. A repeat diagnostic procedure was necessary in 11 (5.0%) of the patients with a repeat pleural procedure changing the ultimate diagnosis in 8 (3.7%) of patients (Table 5). Four of these patients had atypical mesothelial changes identified on their initial MT result and 6 of the 8 patients ultimately had mesothelioma diagnosed as a result of the repeat pleural procedure.

We found that pleural nodularity identified on endoscopy had good sensitivity (76%) and specificity (91%) for malignancy in our cohort (Table 4). Pleural nodularity was also significantly better detected by MT than CT scan (*p* < 0.001). Other endoscopic findings such as pleural thickening, adhesions, pleural plaques, or calcified pleural plaques were not found to be highly predictive.

### 3.2. Safety (Table 6)

Of the 218 outpatient MT and IPC insertions performed, only two patients (0.9%) required hospital admission after procedure. One patient had significant enlarging pneumothorax requiring connection to suction while the other patient had a syncopal event after procedure. Both were discharged after a short hospital stay. A total of four patients required administration of a reversal agent during the procedure for difficulties resulting from conscious sedation; three of these patients received below average doses of sedation. IPC related complications include 8 patients (3.7%) with nondraining IPC requiring intervention and 6 patients (2.8%) who developed pleural infection. The IPC remained in place for a median of 34 days after the procedure. Tumor seeding along the MT and IPC tract was noted in 4 patients (1.8%); all 4 of these patients had a diagnosis of mesothelioma. There was no procedure associated mortality.

## 4. Discussion

This large Canadian cohort adds further evidence that outpatient MT can be performed safely and effectively. Furthermore, our outcomes are comparable to prior reports in this regard [3, 7–9]. However, there were several important differences between our work and previously published reports.

We did not exclude patients on the basis of performance status alone. In fact, 33.5% of patients who had this procedure as an outpatient had an ECOG greater than two. We believe there are several important factors that contributed to the safe completion of the procedure in our patients. First, this is both a diagnostic and therapeutic procedure, and most patients feel better after completion than they did at the time of initial assessment. Second, our patients underwent moderate conscious sedation, similar to the doses administered for bronchoscopy in most Canadian centers, rather than general anesthesia. By avoiding excess sedation, patients were able to breathe spontaneously and became fully awake quickly after the procedure was complete. Third, adequate observation time is important to assess for complications. For example, some patients may have a small pneumothorax that may be due to either a trapped lung, small alveolar pleural fistula, or procedure related. We found that if the pneumothorax had not enlarged after two hours of observation, then they were safe to be discharged home and reviewed in clinic in two weeks. Only one patient in our cohort required a short hospitalization for an enlarging pneumothorax that was captured by the two-hour follow-up chest X-ray. Fourth, IPC follow-up and care by home care nursing services are important to avoid hospital visits. Home care nursing has contact information for our clinic and is able to easily obtain troubleshooting assistance. If the concerns are not easily addressed over the phone, an extra clinic follow-up visit can be arranged. Also patients in further rural areas can be treated at home due to home care services. We feel this arrangement is vital to the safety and efficacy of our outpatient program.

The complications experienced were generally minor and the low rates were comparable to previously published data [4]. No patients died from a complication related to procedure. In our institution, although thoracic surgeons and anesthesiologists are available if needed, we did not require their assistance for any case. We do believe that having surgical and anesthesia assistance available in the hospital can be valuable in the event of an unexpected complication; however, they are not required to be present at the time of the procedure.

Pleural nodularity was an excellent predictor of malignancy with a positive predictive value of 92.5% and negative predictive value of 72.3%. The sensitivity was 76% and specificity 91% (Table 4). In addition, MT was also better at detecting pleural nodularity than CT scans. This is an important finding, because the detection of nodularity may guide method and location of diagnostic testing.

NSP and reactive mesothelial changes were the most common nonmalignant pathological diagnosis in our cohort (34%). The follow-up of this group of patients is an evolving area of interest. Prior reports have suggested that between 3.5 and 12% of patient with this finding will end up with a diagnosis of pleural malignancy generally made within one year of follow-up, particularly mesothelioma [10–12]. In our group of 74 patients with NSP or reactive mesothelial changes, a total of 4 (5.4%) had a diagnosis of malignancy made with another repeat diagnostic procedure within one year. Only one of the four patients was given a diagnosis of mesothelioma. The other patients were found to have non-small-cell lung cancer, stage III thymoma with lung invasion, and non-Hodgkin's lymphoma, respectively. Conversely, patients with atypical mesothelial changes on pathology were more likely to be eventually diagnosed with malignancy. Out of a total of 9 patients with this finding, 4 patients had a repeat diagnostic procedure demonstrating malignancy and the diagnosis was mesothelioma in all 4 cases. Our results suggest that while close radiographic and clinical follow-up may be appropriate for those with NSP or reactive mesothelial changes, patients with atypical mesothelial changes should have definitive repeat biopsy performed.

In our cohort of patients undergoing both MT with IPC insertion, the median time to catheter removal was 34 days. In a review of IPC insertions without MT, the median time to catheter removal was reported to be between 44 and 60 days [13]. We believe the pleural biopsies and the minimal bleeding incurred as a result may be responsible for promoting inflammation in the pleural space, which may in turn lead to earlier removal than with IPC placement alone. If MT is associated with earlier IPC removal, there may be additional savings of healthcare associated costs by reducing need for home care nursing services and access to healthcare. Further studies are needed to confirm this finding, and comparative cost analyses are needed to establish if combining both procedures would be cost-saving when compared to IPC alone.

Our study has several limitations. Our follow-up period was a minimum of two years, which may limit our conclusions as to the final outcomes of patients with NSP. However, prior data suggests that one-year follow-up was sufficient and is consistent with our cohort [10]. Secondly, no conclusions concerning cost-savings can be drawn from our study. A formal cost analysis comparing outpatient and VATS would have to be performed. Thirdly, our study population is likely different from patients referred to a thoracic surgeon who may manage inpatient cases, as our patients were able to arrive and be discharged as an outpatient. In addition, thoracic surgeons are referred more complicated pleural disease. Consequently, not all pleural disease patients are appropriate for MT.

## 5. Conclusions

MT combined with IPC placement can be used safely and successfully as an outpatient procedure. Particularly in Canada, where the healthcare system is publically funded and resource allocation is carefully scrutinized, outpatient MT may be a convenient and potentially cost-saving alternative to inpatient operative procedures, although this requires further study. We believe that MT should be a key component of pleural disease treatment programs.

## Figures and Tables

**Table 1:** Patient characteristics.

Characteristic	*N* = 218
Age (mean ± SD)	68.2 ± 12.5

*Sex (n, %) *	
Male	114 (52.3)
Female	104 (47.7)

*ECOG* ^*∗*^ * performance status (n, %) *	
1	39 (17.9)
2	106 (48.6)
3	64 (29.4)
4	9 (4.1)

BDI^†^ (mean ± SD)	3.7 ± 1.3
TDI^‡^ (mean ± SD)	5.6 ± 2.0

*Previous thoracentesis (n, %) *	
0	32 (14.7)
1	133 (61.3)
>1	52 (24.0)

*CT imaging (n, %) *	
Single effusion alone	19 (9.9)
Bilateral effusion	46 (24.0)
Pleural nodularity	61 (31.8)
Pleural thickening	79 (41.1)
Adenopathy	57 (29.7)
Pulmonary nodules	51 (26.6)
Mass	33 (17.2)
Calcified pleural plaques	14 (7.3)
No recent CT	26 (11.9)

Indwelling pleural catheter already in place at time of procedure (*n*, %)	8 (3.7)

^*∗*^Eastern Cooperative Oncology Group. ^†^Baseline dyspnea index. ^‡^Transition dyspnea index.

**Table 2:** Procedural details.

Procedural detail	

Mean procedure time in minutes (mean ± SD)	46.7 ± 13.6

*Sedation/analgesia*	
Midazolam dose in mg (mean ± SD)	2.2 ± 0.6
Fentanyl dose in mcg (mean ± SD)	92 ± 40.4

Pleural fluid removed at time of procedure (mL ± SD)	1513 ± 1054

*Indwelling pleural catheter *	
Preexisting catheter (*n*, %)	8 (3.7)
Catheter inserted at time of procedure (*n*, %)	210 (96.3)

*Endoscopic findings (n, %)*	
Normal	10 (4.6)
Nodular abnormalities	106 (48.6)
Pleural thickening	128 (58.7)
Adhesions	95 (43.6)
Pleural plaques	24 (11.0)
Erythema/inflammatory changes alone	6 (2.8)

**Table 3:** Pathology results.

Pathology	*N* (%)
*Malignancy*	130 (59.6)
Non-small-cell lung cancer	47 (21.6)
Adenocarcinoma	42 (19.3)
Squamous	3 (1.4)
Large cell	2 (0.9)
Mesothelioma	20 (9.2)
Epithelioid	13 (6.0)
Sarcomatoid	3 (1.4)
Biphasic	4 (1.8)
Others	63 (28.9)
Small-cell lung cancer	2 (0.9)
Breast	27 (12.4)
Renal	2 (0.9)
Ovarian adenocarcinoma	7 (3.2)
Papillary serous	4 (1.8)
Melanoma	2 (0.9)
Colorectal	1 (0.5)
Chronic lymphocytic leukemia	4 (1.8)
Lymphoma/lymphoproliferative	2 (0.9)
Parotid	1 (0.5)
Sarcoma	2 (0.9)
Thyroid	1 (0.5)
Laryngeal	2 (0.9)
Esophageal	1 (0.5)
Vulvar	1 (0.5)
Carcinoma unknown primary	4 (1.8)

Nonspecific pleuritis	64 (29.4)

Reactive mesothelial changes	10 (4.6)

Atypical mesothelial changes	9 (4.1)

Granulomatous pleuritis	3 (1.4)

Eosinophilic pleuritis	1 (0.5)

Hematoma	1 (0.5)

**Table 4:** Performance characteristics of endoscopic findings related to malignancy, in 218 patients undergoing medical thoracoscopy for diagnostic purposes.

Endoscopy findings	Sensitivity (95% CI)	Specificity (95% CI)	PPV (95% CI)	NPV (95% CI)
Pleural nodules	76 (52.3–65.7)	91 (82.6–95.8)	92.5 (85.2–96.4)	72.3 (62.9–80.2)
Pleural thickening	55.8 (46.8–64.5)	37 (27.2–48)	56.3 (47.2–64.9)	36.7 (26.9–47.5)
Adhesions	36.4 (28.3–45.4)	46.1 (35.6–56.9)	49.5 (39.1–59.9)	33.3 (57.5–74.8)
Erythema	18.6 (12.5–26.6)	73 (62.4–81.6)	50 (35.4–64.5)	38.2 (31–46)
Calcified pleural plaques	3.9 (1.4–9.3)	93.3 (85.4–97.2)	45.5 (18.1–75.4)	40.1 (33.4–47.1)
Pleural plaques	6.2 (2.9–12.2)	94.4 (86.8–97.9)	61.5 (32.3–84.9)	41 (51.9–65.8)

**Table 5:** Postprocedure details.

*Postprocedure details*	
Days before catheter removal (median days, IQR)	34 (14.0–81.5)
Repeat diagnostic procedure performed (*n*, %)	11 (5.0)
Repeat diagnostic pleural procedure performed (*n*, %)	9 (4.1)

*Instances that repeat pleural procedure altered diagnosis (n, %)*	
New diagnosis made	8 (3.7)
Mesothelioma	6 (2.8)
Non-small-cell lung cancer	1 (0.5)
Thymoma	1 (0.5)

**Table 6:** Complications.

Complication	*N* = 24
Blocked catheter requiring intervention	8
Pleural infection	6
Tumor growth at catheter site	4
Sedation reversal agent administered	4
Admission required after procedure	2
